# Biomarker Differences in Post‐acute Sequelae of COVID‐19 Patients Compared to Controls

**DOI:** 10.1002/alz.089401

**Published:** 2025-01-09

**Authors:** Allal Boutajangout, Jennifer A. Frontera, Arjun V. Masurkar, Ludovic Debure, Wajiha Ahmed, Alok Vedvyas, Jon Links, Li Jiang, Sujata Thawani, Zhe Sun, Mobeena Ghuman, Elizabeth Pirraglia, Rebecca A Betensky, Yongzhao Shao, Yulin Ge, Thomas Wisniewski

**Affiliations:** ^1^ NYU Grossman School of Medicine, New York, NY USA; ^2^ Center for Cognitive Neurology, New York, NY USA; ^3^ Center for Cognitive Neurology, New York University Langone Health, New York, NY USA; ^4^ NYU Grossman School of Medicine, New York, NY USA; ^5^ NYU Alzheimer's Disease Research Center, New York, NY USA

## Abstract

**Background:**

Cognitive impairment is a major symptom among patients with post‐acute sequelae of COVID‐19; the underlying pathogenesis is unknown. This impairment may be associated with changes in the level of plasma biomarkers of neurodegeneration and neuroinflammation.

**Method:**

Plasma samples were collected from COVID‐19 patients (Covpos, median 724 days from index SARS‐CoV‐2 infection), and from non‐COVID‐19 controls (Covneg; no history of SARS‐CoV‐2 infection and negative SARS‐CoV‐2 nucleocapsid antibody). All patients underwent neuropsychological testing using the Uniform Data Set Version‐3 Cognitive Battery. Patients were coded as having cognitive impairment (Cogabnormal) based on neuropsychological testing (≥1 abnormal test result) or cognitively normal (Cognormal) (normal performance). Plasma biomarker assays Aβ40, Aβ42, NFL, and GFAP were measured using an N4PE kit. pTau181 V2 was measured on the pTau181 kit, using Simoa HD‐X technology that employs sensitive immunoassays with a limit of detection under 100 fg/ml. Multiple linear regression models were fit for each biomarker, with covariates of binary cognitive status and COVID‐19 and their interaction, and age, sex and education. If the interaction term was not statistically significant (p<0.05), it was removed from the model. No adjustments were made for multiple comparisons in this exploratory analysis.

**Results:**

A total of 131 subjects were enrolled: Covpos / Cognormal (n=61), Covpos/Cogabnormal (n=46), Covneg/Cognormal (n=11), and Covneg/Cogabnormal (n=13). The Cog‐Covid interaction terms were significant for Aβ42 and pTau181/Aβ42 ratio. The mean levels of Aβ42 in Covpos/Cogabnormal is significantly lower than in Covpos/Cognormal (‐1.05, 95%CI[‐1.74,‐0.36], p=0.003), and trending lower in Covneg/Cogabnormal (‐1.12, 95%CI [‐2.26,0.01], p=0.05), and not significantly different in Covneg/Cognormal. Older subjects had a higher pTau181/Aβ42 ratio (0.05, 95%CI[0.03,0.07], p<0.001). The levels of pTau181/Aβ42 ratio in Covpos/Cogabnormal were significantly higher than in Covneg/Cogabnormal (1.68, 95%CI [0.60,2.76], p=0.003), and in Covpos/Cognormal (0.92, 95%CI[0.27,1.58], p=0.007) and not significantly different in Covneg/Cognormal. Other biomarkers showed no significant differences between any of the groups.

**Conclusion:**

Biomarker levels of Aβ42 and pTau181/Aβ42 ratio in COVID‐19 plasma samples were significantly different in COVID‐19 patients with cognitive impairment compared to COVID‐19 patients with normal cognition. Additionally, among all patients with cognitive impairment, those with COVID‐19 had significantly higher ptau181/Aβ42, suggestive of an Alzheimer’s‐type neurodegenerative process.

